# Optimising exercise intensity for gut health: Effect on microbiota composition, barrier integrity and inflammation in male Wistar rats

**DOI:** 10.1113/EP092970

**Published:** 2025-12-28

**Authors:** Nova Sylviana, Putri Karisa, Nur Faizah Romadona, Imam Megantara

**Affiliations:** ^1^ Physiology Division, Department of Biomedical Sciences, Faculty of Medicine Universitas Padjadjaran Bandung Indonesia; ^2^ Physiolgy Molecular Laboratory, Biology Activity Division, Central Laboratory Universitas Padjadjaran Sumedang West Java Indonesia; ^3^ Center of Sport Science, Wellness, and Longevity, Graduate School Universitas Padjadjaran Bandung West Java Indonesia; ^4^ Doctoral Program in Medical Science, Faculty of Medicine Universitas Padjadjaran Bandung Indonesia; ^5^ Faculty of Medicine Universitas Pendidikan Indonesia Bandung West Java Indonesia; ^6^ Microbiology Division, Department of Biomedical Sciences, Faculty of Medicine Universitas Padjadjaran Bandung Indonesia

**Keywords:** *Akkermansia muciniphila*, exercise, *Faecalibacterium prausnitzii*, gut barrier, gut microbiota, inflammation

## Abstract

Exercise influences gut microbiota composition and intestinal permeability, but the optimal intensity for maintaining gut health remains unclear. This study investigates the effects of different exercise intensities on abundance of some gut microbiota, epithelial barrier integrity and inflammatory markers. Male Wistar rats were randomly assigned to control (*n* = 5), low‐intensity (10 m/min, *n* = 5), moderate‐intensity (20 m/min, *n* = 5) and high‐intensity (30 m/min, *n* = 5) exercise groups, exercising five times per week for 8 weeks. The relative abundance of *Akkermansia muciniphila*, *Faecalibacterium prausnitzii* and *Escherichia coli* was quantified using qPCR, whilst mRNA levels of zonula occludens‐1 (ZO‐1) and interleukin‐6 (IL‐6) were assessed as markers of barrier function and inflammation. *A. muciniphila* and *F. prausnitzii* abundance increased at moderate exercise intensity (*P *< 0.005), but decreased at high intensity (*P* = 0.0019), whereas *E. coli* rose sharply at high intensity (*P *< 0.001). ZO‐1 expression was higher at moderate (β = 0.30, 95% CI 0.195–0.405, *P *< 0.001) and high intensity (β = 0.60, 0.495–0.705, *P *< 0.001), but not at low intensity (β = 0.10, −0.005–0.205, *P *> 0.05). IL‐6 increases similarly (moderate β = 0.50, 0.247–0.753, high β = 1.10, 0.847–1.353, both *P *< 0.001). In conclusion, moderate‐intensity exercise enhanced beneficial microbiota and epithelial barrier integrity, whereas high‐intensity exercise promoted *E. coli* proliferation and IL‐6‐mediated inflammation, underscoring a dose‐dependent, bidirectional regulation of the gut epithelial interface.

## Abstract (highlights)

- **What is the central question of this study?**
  What are the effects of different exercise intensities on abundance of some gut microbiota, epithelial barrier integrity and inflammatory markers?
- **What is the main finding and its importance?**
  Overall, these findings highlight the importance of considering exercise intensity in the context of gut health. Although exercise is generally recognised as beneficial, too high an intensity can disrupt microbiota balance and weaken gut barrier function, increasing the risk of inflammation. In contrast, moderate‐intensity exercise appears to be the optimal choice for maintaining gut health and preventing inflammatory disorders.

## 1 INTRODUCTION

The gastrointestinal tract harbours trillions of microorganisms collectively referred to as the gut microbiota, and they play a crucial role in metabolism, immunity and maintenance of intestinal barrier integrity (Sender et al., 2016). The balance of microbial populations influences host health, and dysbiosis (an imbalance in the microbiota) has been associated with metabolic disorders, inflammatory bowel diseases and systemic inflammation (Turnbaugh et al., 2006). Various factors, including diet, antibiotics and exercise, influence gut microbiota composition and function (David et al., 2014; Zarrinpar et al., 2014).

Exercise is widely recognised for its beneficial effects on systemic health, including improvements in metabolic function, cardiovascular health and immune responses (Gleeson et al., 2011). Recent studies suggest that exercise also plays a critical role in modulating the gut microbiota, with both positive and negative outcomes depending on the intensity and duration of physical activity (Barton et al., 2018). Regular, moderate‐intensity exercise has been shown to enhance microbial diversity, promoting the growth of beneficial bacteria such as *Akkermansia muciniphila* and *Faecalibacterium prausnitzii*, which contribute to gut homeostasis and reduce systemic inflammation (Monda et al., 2017; O'Sullivan et al., 2015). Conversely, excessively high‐intensity exercise may negatively affect gut barrier integrity, increase gut permeability and elevate systemic inflammatory markers, potentially leading to gastrointestinal distress (Clark & Mach, 2016; Karl et al., 2017).

The integrity of the gut barrier is largely maintained by tight junction proteins, such as zonula occludens‐1 (ZO‐1), which regulate the permeability of intestinal epithelial cells and prevent the translocation of bacteria and endotoxins into the bloodstream (Turner, 2009). Increased expression of ZO‐1 may arise from distinct underlying mechanisms. In some contexts, it reflects a physiological adaptation designed to reinforce the intestinal barrier and limit paracellular permeability, thereby preserving mucosal integrity. Conversely, a pronounced upregulation of ZO‐1, particularly at elevated levels, may signify a compensatory response to epithelial injury or cellular stress rather than an actual enhancement of barrier function (Leppkes & Neurath, 2020). Increased gut permeability allows bacterial lipopolysaccharides (LPS) to enter the circulation, triggering an immune response that elevates interleukin‐6 (IL‐6). Although IL‐6 plays a role in muscle repair and immune modulation post‐exercise, sustained elevations are associated with systemic inflammation and metabolic dysfunction (Pedersen & Febbraio, 2008).

However, the mechanisms linking different exercise intensities with gut microbial composition, barrier integrity and inflammatory responses are not fully understood. Understanding these interactions is critical for designing training regimens that support both performance and overall health. Therefore, this study aims to evaluate the impact of different exercise intensities on gut microbiota diversity, ZO‐1 expression and IL‐6 levels in male Wistar rats, to identify the optimal exercise intensity for maintaining gut homeostasis and reducing inflammation.

## 2 METHODS

### 2.1 Ethical approval

The research protocols were approved by the Research Ethics Committee for Animal Use of Faculty of Medicine, Universitas Padjadjaran, Indonesia (No.315/UN6.KEP/EC/2024). This study was conducted according to the Guide for the Care and Use of Laboratory Animals in accordance with AARIVE 2.0 guidelines (du Sert et al., 2020). The rats were housed at the Laboratory Science of Universitas Padjadjaran (Bandung, Indonesia). Experimental analyses were carried out at the Biology Activity Laboratory, Central Laboratory, Universitas Padjadjaran (Sumedang, Indonesia).

### 2.2 Animals

A total of 20 male Wistar rats, aged 8–12 weeks, and weighing 200–250 g, were obtained from PT Biofarma (Parongpong, West Java, Indonesia). Each of the five rats was placed in a 45 × 30 × 25 cm cage, provided with a bed of fine sawdust, at a temperature of 22 ± 2°C, humidity of 50–60% and a light/dark cycle of 12/12 h and had ad libitum access to standard laboratory chow and water. The standard normal diet pellets were obtained from Surya Sains Indonesia Company (Bandung, Indonesia).

Following the acclimatisation period, the rats were randomly assigned into four groups: control group (sedentary, *n* = 5), low intensity (*n* = 5), moderate intensity (*n* = 5) and high intensity aerobic exercise (*n* = 5). The sample was determined using Mead's resource equation method. The calculation is done by the formula: DF = *N* − *k* = *kn* − *k* = *k*(*n* − 1), where DF is degrees of freedom [the DF in the formula is replaced with the minimum (*n* = 10) and maximum (*n* = 20)], *N* is total number of subjects, *k* is number of groups and *n* is number of subjects per group. This study consisted of three groups, so the minimum number of animals/group was four. To avoid unexpected events, 10% was added to the group, so the total sample was five rats per group.

The sample inclusion criteria of this study were as follows: (1) male Wistar rats with physically healthy conditions with no abnormalities or disease, especially those related to the digestive systems, (2) 8 weeks of age, and (3) a body weight of around 150–250 g. Additional criteria for sample selection included: (4) fresh faeces preserved at a temperature of 2°C and examined within 24–48 h after the extraction. The exclusion criteria of this in vivo study were: (1) rats with a significant decrease in body weight during the adaptation process, (2) rats that showed a reduction in hair quantity or alterations in the colour of the sclera and mouth, and (3) rats that refused the exercise protocol after the adaptation process.

### 2.3 Exercise protocol

The treadmill exercise protocol was designed to mimic different levels of physical exertion whilst maintaining consistency in duration across groups. The rats were divided into the following groups: (1) control (sedentary group): no treadmill exercise was performed; (2) low‐intensity exercise: 10 m/min for 30 min/day; (3) moderate‐intensity exercise: 20 m/min for 30 min/day; and (4) high‐intensity exercise: 30 m/min for 30 min/day. Each exercise session was conducted under supervision, and treadmill speeds were adjusted gradually to allow adaptation in the initial week. The exercise intensity was determined based on lactate accumulation levels and prior research findings (Gunadi et al., 2020; Lesmana et al., 2016; National Research Council, 2011).

### 2.4 Sample collection and tissue processing

After the 8‐week intervention, rats were terminated using a high dose of anaesthetic (100 mg/kg of ketamine and 10 mg/kg of xylazine) via intraperitoneal injection. All specimens were collected exclusively at this terminal time point. Immediately *post mortem*, the distal colon was exposed under aseptic conditions. Luminal contents from the distal colon (hereafter referred to as faecal material for microbiota analysis) were gently expressed into sterile cryovials, snap‐frozen on dry ice, and stored at −80°C until DNA extraction for qPCR‐based quantification of *A. muciniphila*, *F. prausnitzii* and *Escherichia coli*. Colon tissue segments adjacent to the sampled region were rinsed in cold phosphate‐buffered saline (PBS), blotted dry, snap‐frozen and stored at −80°C for RNA extraction and gene‐expression analyses (ZO‐1, IL‐6). No opportunistic (cage) faecal collections were performed, and no pre‐ or mid‐intervention samples were obtained; thus, all microbiota and molecular readouts represent the post‐intervention terminal endpoint in each intensity group (Control, Low, Moderate, High).

### 2.5 Sample analysis faecal microbiome DNA extraction quantification and sequencing

Quantification of microbiota DNA was conducted to detect the presence and determine the concentration of microbiota DNA prior to amplification and qPCR analysis. The 16S rDNA gene sequencing procedure was carried out at the Central Laboratory of Universitas Padjadjaran (Sumedang, Indonesia). Microbiota samples were taken from the faeces of 20 rats, collected post‐termination immediately from the distal colon to avoid environmental contamination. Samples were preserved at 2°C and processed within 24–48 h.

DNA extraction was performed using a Maxwell RSC 48 (Promega Corp., Madison, WI, USA) and a Maxwell RSC Fecal Microbiome DNA Kit (Promega), following the manufacturer's protocol. DNA concentration was measured using Quantifor Thermo Scientific Multiskan GO (Thermo Scientific, Waltham, MA, USA) with the dsDNA detection method, and only samples with a concentration ≥2.77 ng and purity ≥1.70 (*A*_260_/*A*_280_) were included.

For qPCR, amplification of bacterial taxa (e.g., *E. coli*, *A. muciniphila*, *F. prausnitzii*) was performed using species‐specific primers (Table 1). Each reaction consisted of 1 µL template DNA mixed with 3.2 µL nuclease‐free water, 5 µL qPCR mix, 0.4 µL forward primer and 0.4 µL reverse primer, and was run in an AriaMx Real‐Time PCR instrument (Agilent Technologies, Santa Clara, CA, USA) using kits from Toyobo Corporation (Osaka, Japan).

**TABLE 1:** Primer sequences.

Gene or bacterium	Species	Primer	Sequence
*E. coli*	Rat	Forward	CACGAACTGAACTGGCAGA
		Reverse	CATTACGCTGCGATGGAT
*F. prausnitzii*	Rat	Forward	GGAGGAAGAAGGTCTTCGG
		Reverse	AATTCCGCCTACCTTCGCACT
*A. muciniphila*	Rat	Forward	CAGCACGTGAAGGTGGGGAC
		Reverse	CCTTGCGGTTGGCTTCAGAT
ZO‐1	Rat	Forward	CGGTCCTCTGAGCCTGTAAG
		Reverse	GGATCTACATGCGACGACAA
IL‐6	Rat	Forward	TCCTACCCCAACTTCCAATGCTC
		Reverse	TTGGATGGTCTTGGTCCTTAGCC
GAPDH	Rat	Forward	GTTACCAGGGCTGCCTTCTC
		Reverse	GATGGTGATGGGTTTCCCGT

Relative quantification of each bacterial group was normalised against total bacterial 16S rRNA gene abundance (universal 16S primers) to correct for inter‐sample variation in DNA input. The comparative *C*_t_ (Δ*C*_t_) method was applied, where Δ*C*_t_ = *C*_t_(target) − *C*_t_(total 16S). Relative abundance was calculated using the $2^{-\Delta C_{t}}$ formula, and values were expressed as a percentage of total bacteria by dividing each target abundance by the sum of all quantified bacteria × 100. Group‐level differences were then compared across experimental conditions.

### 2.6 RNA isolation and semi‐quantitative real‐time PCR

The gene expression levels of ZO‐1 (tight junction marker) and IL‐6 (inflammatory marker) were measured using RT‐qPCR with glyceraldehyde‐3‐phosphate dehydrogenase (GAPDH) as a normalisation housekeeping gene. PCR was performed by mixing 5 µL 2× Sensifast SYBR mix, 0.4 µL primer forward, 0.4 µL primer reverse, 0.2 µL RNase inhibitor, 0.1 µL reverse transcriptase enzyme, 2 µL RNA template and 1.9 µL DEPC for a total volume 10 µL (SensiFAST SYBR Lo‐ROX One‐Step Kit, Bioline, Memphis, TN, USA) (SensiFast, 2019). RT‐PCR was performed using AriaMx SYBR Real‐time PCR technology (Agilent Technologies, Santa Clara, CA, USA). The primer sequences were mouse‐based and designed using the Primer‐BLAST tool (NCBI; http://www.ncbi.nlm.nih.gov/Genbank/index.html; Integrated DNA Technologies, Coralville, IA, USA) (Table 1). The relative expression was determined using the $2^{-\Delta \Delta C_{t}}$ method and normalised using GAPDH as the reference (Livak & Schmittgen, 2001).

### 2.7 Statistical analysis

All statistical analyses were performed using SPSS v26 (IBM Corp., Armonk, NY, USA) and GraphPad Prism v9 (GraphPad Software, Boston, MA, USA). One‐way ANOVA was used to compare microbiota abundance and gene expression fold‐change levels across groups, and Tukey's HSD *post hoc* test was applied to identify pairwise differences. Effect sizes (Cohen's *d*) were calculated to determine the magnitude of differences between exercise intensities. In addition, linear regression models were fitted with fold‐change as the dependent variable and exercise intensity (no exercise, low, moderate, high) as a categorical predictor, with the No Exercise group specified as the reference category. In this parameterisation, the intercept represents the mean fold‐change in the No Exercise group, whereas the β coefficients for the exercise conditions represent the mean differences in fold‐change compared with the No Exercise group. Statistical significance was set at *P* < 0.05, and data were expressed as means ± standard deviation (SD).

## 3 RESULTS

### 3.1 The microbiota, barrier integrity and inflammation across exercise groups

**FIGURE 1:**
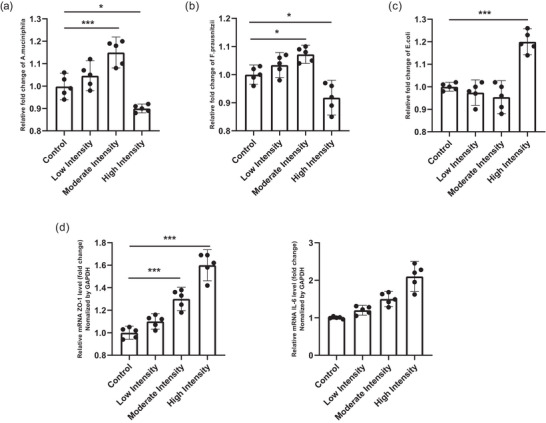
Relative fold change of *Akkermansia muciniphila*, *Faecalibacterium prausnitzii* and *Escherichia coli*, and relative mRNA expression of ZO‐1 and IL‐6 across exercise intensities (control, low, moderate, high). (a) One‐way ANOVA showed a significant overall effect for *A. muciniphila* (*P* < 0.05). Relative to control, levels increased at moderate intensity (*P* < 0.001) but decreased at high intensity (*P* = 0.019). (b) For *F. prausnitzii*, ANOVA indicated a significant group effect (*P*< 0.05). Relative to control, moderate intensity showed an increase (*P* = 0.028), whereas high intensity showed a decrease (*P* = 0.011). (c) For *E. coli*, ANOVA revealed a significant group effect (*P* < 0.05), with a marked increase at high intensity versus control (*P* < 0.001). (d) ZO‐1 expression exhibited a significant overall effect by ANOVA (*P*< 0.05) and was higher at both moderate and high intensities (both *P* < 0.001 vs. control). (e) IL‐6 expression also showed a significant overall effect (*P* < 0.05), increasing at moderate (*P* = 0.004) and high intensities (*P* < 0.001) compared with control. Data are presented as mean ± 95% CI (*n* = 5 per group). Group differences were tested by one‐way ANOVA followed by Tukey's *post hoc* test. Asterisks denote significance versus control: *P < 0.05, **P < 0.01, ***P < 0.001.

Integrated analysis of gene expression and gut microbiota revealed a coordinated response to varying exercise intensities (Figure 1). ZO‐1 expression demonstrated a progressive upregulation from the control to the high‐intensity group, indicating that increasing exercise intensity enhances epithelial barrier integrity through tight‐junction protein regulation. Similarly, IL‐6 expression showed a graded rise, reflecting an intensity‐dependent inflammatory response, with high‐intensity exercise eliciting the strongest cytokine activation.

These molecular changes were paralleled by distinct shifts in gut microbiota composition (Figure 1). *A. muciniphila* and *F. prausnitzii*, both beneficial commensals associated with mucosal health, increased under low‐to‐moderate intensity but decreased at high intensity, suggesting that moderate exercise optimises gut barrier–microbiota crosstalk, whereas excessive training induces stress‐related dysbiosis. Conversely, *E. coli* abundance rose sharply at high intensity, indicating potential pro‐inflammatory microbial activity consistent with elevated IL‐6 expression. Collectively, these findings suggest that moderate‐intensity exercise promotes a balanced gut–barrier interaction by upregulating ZO‐1 and maintaining beneficial microbiota, whilst high‐intensity training disrupts this equilibrium, amplifying IL‐6‐mediated inflammation and favouring opportunistic microbial expansion. This highlights a dose‐dependent dual effect of exercise intensity on the gut–epithelial axis (Figure 1).

These observed patterns were statistically supported by *post hoc* analyses summarised in Table 2. The *post hoc* Tukey test confirmed that the changes in both microbial composition and gene expression were significant across exercise intensities. Specifically, *A. muciniphila* and *F. prausnitzii* showed significant increases in the moderate‐intensity group compared to control, followed by marked decreases at high intensity, whereas *E. coli* exhibited a significant elevation under high‐intensity exercise (Table 2). Consistent with these microbial alterations, ZO‐1 expression was significantly upregulated at both moderate and high intensities, indicating enhanced epithelial integrity, whilst IL‐6 expression rose progressively with intensity, reflecting the activation of inflammatory pathways (Table 3). These statistical outcomes reinforce the bidirectional, intensity‐dependent regulation between the gut microbiota and epithelial gene expression observed in the experimental findings.

**TABLE 2:** Comparison of gut microbiota expression across different exercise intensity groups in faecal samples of rats (*n* = 20).

Biomarker	Comparison	Mean difference	SE	95% confidence interval	*P*
*A. muciniphila*	Control vs. low‐intensity	−0.048	0.029	−0.1317 to 0.357	0.385
	Control vs. moderate‐intensity	−0.152		−0.2357 to −0.0683	<0.001^***^
	Control vs. high‐intensity	0.098		0.0143 to 0.1817	0.019^*^
	Low vs. moderate‐intensity	−0.104		−0.1877 to −0.0203	0.013^*^
	Low vs. high‐intensity	0.146		0.0623 to 0.2297	<0.001^***^
	Moderate vs. high‐intensity	0.250		0.1663 to 0.3337	<0.001^***^
*F. prausnitzii*	Control vs. low‐intensity	−0.034	0.023	−0.0991 to 0.0311	0.463
	Control vs. moderate‐intensity	−0.072		−0.1371 to −0.0069	0.028^*^
	Control vs. high‐intensity	0.082		0.0169 to 0.1471	0.011^*^
	Low vs. moderate‐intensity	−0.038		−0.1031 to 0.271	0.370
	Low vs. high‐intensity	0.116		0.0509 to 0.1811	<0.001^***^
	Moderate vs. high‐intensity	0.154		0.0889 to 0.2191	<0.001^***^
*E.coli*	Control vs. low‐intensity	0.026	0.028	−0.0547 to 0.1067	0.794
	Control vs. moderate‐intensity	0.046		−0.0347 to 0.1267	0.390
	Control vs. high‐intensity	−0.200		−0.2807 to −0.1193	<0.001^***^
	Low vs. moderate‐intensity	0.020		−0.0607 to 0.1007	0.892
	Low vs. high‐intensity	−0.226		−0.3067 to −0.1453	<0.001^***^
	Moderate vs. high‐intensity	−0.246		−0.3267 to −0.1653	<0.001^***^

^*^ *P* < 0.05, ^***^ *P* < 0.001.

**TABLE 3:** Comparison of ZO‐1 and IL‐6 expression across different exercise intensity groups in colon tissue samples of rats (*n* = 20).

Biomarker	Comparison	Mean difference	SE	95% confidence interval	*P*
ZO‐1	Control vs. low‐intensity	−0.100	0.496	−0.2419 to 0.0419	0.223
	Control vs. moderate‐intensity	−0.300		−0.4419 to −0.1581	<0.001^***^
	Control vs. high‐intensity	−0.600		−0.7419 to −0.4581	<0.001^***^
	Low vs. moderate‐intensity	−0.200		−0.3419 to −0.0581	0.005^*^
	Low vs. high‐intensity	−0.500		−0.6419 to −0.3581	<0.001^***^
	Moderate vs. high‐intensity	−0.300		−0.4419 to −0.1581	<0.001^***^
IL‐6	Control vs. low‐intensity	−0.200	0.119	−0.5416 to 0.1416	0.368
	Control vs. moderate‐intensity	−0.500		−0.8416 to −0.1584	0.004^*^
	Control vs. high‐intensity	−1.100		−1.4416 to −0.7584	<0.001^***^
	Low vs. moderate‐intensity	−0.300		−0.6416 to 0.416	0.096
	Low vs. high‐intensity	−0.900		−1.2416 to −0.5584	<0.001^***^
	Moderate vs. high‐intensity	−0.600		−0.9416 to −0.2584	<0.001^***^

^*^ *P* < 0.05, ^**^ *P* < 0.01, ^***^ *P* < 0.001.

### 3.2 Regression analysis of ZO‐1 and IL‐6 across exercise intensity groups

Regression analysis of ZO‐1 and IL‐6 expression across exercise intensities revealed a clear dose–response pattern that mirrors the microbiota shifts (Table 4 and Figure 1). For ZO‐1, low intensity produced a non‐significant change versus control (β = 0.10, 95% CI −0.005 to 0.205, *P* = 0.061), whereas moderate and high intensities significantly upregulated expression (β = 0.30, 95% CI 0.195 to 0.405, *P* < 0.001; β = 0.60, 95% CI 0.495 to 0.705, *P* < 0.001). IL‐6 showed a parallel intensity‐dependent rise: low intensity was non‐significant (β = 0.20, 95% CI −0.053 to 0.453, *P* = 0.113), whilst moderate and high intensities markedly increased expression (β = 0.50, 95% CI 0.247 to 0.753, *P* < 0.001; β = 1.10, 95% CI 0.847 to 1.353, *P* < 0.001). These molecular responses aligned with the microbiota composition: *A. muciniphila* and *F. prausnitzii* were enriched at moderate intensity, consistent with the observed ZO‐1 upregulation and improved barrier tone, whereas high intensity was accompanied by a decline in these beneficial taxa and a rise in *E. coli*, concordant with the elevation of IL‐6. Taken together, the data indicate that moderate‐intensity aerobic exercise supports a beneficial gut‐epithelial milieu (higher ZO‐1 with commensal enrichment), whilst high‐intensity exercise shifts the axis towards pro‐inflammatory signalling (higher IL‐6) and dysbiosis.

**TABLE 4:** Regression analysis of ZO‐1 and IL9‐6 expression versus exercise intensity.

	No exercise (Control) (*n* = 5)				Low intensity (*n* = 5)				Moderate intensity (n = 5)				High intensity (*n* = 5)			
	β (95% CI)	SE	*t*	*P*	β (95% CI)	SE	*t*	*P*	β (95% CI)	SE	*t*	*P*‐value	β (95% CI)	SE	*t*	*P*
**ZO‐1**	1 [0.926 to 1.074]	0.035	28.513	<0.001^***^	0.1 [−0.005 to 0.205]	0.50	2.016	0.061	0.3 [0.195 to 0.405]	0.050	6.049	<0.001^***^	0.6 [0.495 to 0.705]	0.050	12.097	<0.001^***^
**IL‐6**	1 [0.821 to 1.179]	0.84	11.843	<0.001^***^	0.2 [−0.053 to 0.453]	0.119	1.675	0.113	0.5 [0.247 to 0.753]	0.119	4.187	<0.001^***^	1.1 [0.847 to 1.353]	0.119	9.212	<0.001^***^

*Note*: The Control (no exercise) group is the reference (intercept). β coefficients for Low, Moderate and High intensity represent the adjusted mean difference in fold‐change compared with the No Exercise group. *P*‐value test *H*_0_: β = 0 (no difference from No Exercise). The *P*‐value for the intercept is not interpreted, as a test of *H*_0_: β = 0 is not meaningful for fold‐change. ^*^ *P* < 0.05, ^**^ *P* < 0.01, ^***^ *P* < 0.001.

## 4 DISCUSSION

**FIGURE 2:**
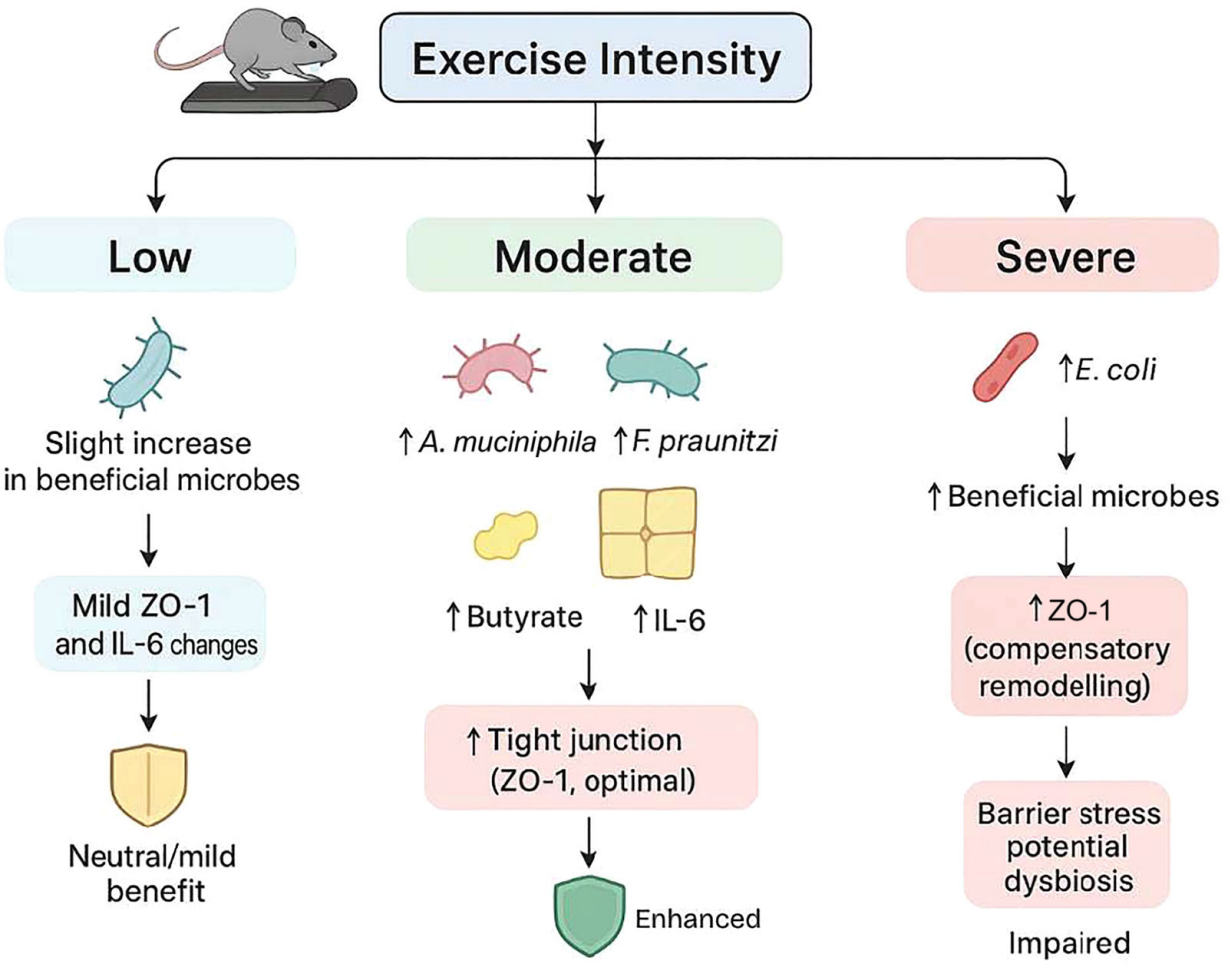
This figure summarises the proposed effects of different exercise intensities on gut microbial shifts and downstream markers of intestinal barrier function and inflammation in the study model. **Low** intensity is associated with a slight increase in beneficial microbes, producing **mild changes in ZO‐1 and IL‐6** and an overall neutral/mild benefit. **Moderate** intensity is associated with increased *Akkermansia muciniphila* and *Faecalibacterium prausnitzii*, accompanied by higher butyrate availability and a controlled IL‐6 response, supporting **optimal tight‐junction integrity (ZO‐1)** and enhanced barrier function. **Severe** intensity is associated with increased *E. coli* and microbial imbalance; although **ZO‐1 may increase as a compensatory remodelling response**, the overall pattern indicates barrier stress with potential dysbiosis and impaired barrier function. Arrows indicate the direction of change. **ZO‐1 = zonula occludens‐1; IL‐6 = interleukin‐6**.

Our findings reinforce the emerging evidence that exercise is a modulator of gut microbiota and intestinal health. Moderate‐intensity exercise significantly increased the relative abundance of *A. muciniphila*, a mucin‐degrading bacterium that stimulates mucus layer renewal and strengthens barrier defence whilst exerting immune‐modulatory effects (Everard et al., 2013; Torquati et al., 2023; Wang et al., 2022). Similarly, *F. prausnitzii*, a major butyrate‐producing bacterium, was enriched under moderate exercise. Butyrate serves as a key energy source for colonocytes, enhances tight junction assembly and suppresses pro‐inflammatory signalling pathways such as NF‐κB (Martín et al., 2023; Torquati et al., 2023; Yang et al., 2021). Thus, the combined enrichment of mucin‐utilising and SCFA‐producing taxa likely contributed to improved gut barrier maintenance and reduced inflammatory tone in the moderate‐intensity group. These results are consistent with previous reports showing that exercise‐induced enrichment of SCFA‐producing bacteria supports intestinal and systemic metabolic health (Keohane et al., 2019; Torquati et al., 2023). To our knowledge, this is amongst the first studies to demonstrate intensity‐dependent microbial and inflammatory shifts using a controlled treadmill protocol in Wistar rats (Figure 2).

Different results were observed in the high‐intensity exercise group, showing a significant increase in *E. coli*. It is important to note that *E. coli* encompasses both commensal strains, which are normal inhabitants of the gut, and pathogenic strains with disease‐causing potential. Our analysis did not distinguish between these subtypes; thus, the observed increase reflects overall *E. coli* abundance. Nevertheless, environmental changes induced by high‐intensity exercise, such as shortened transit time, epithelial stress or altered oxygen gradients, may theoretically promote the expansion of pathogenic *E. coli* strains (Tomasello et al., 2016). Furthermore, changes in digesta transit time due to high‐intensity exercise can affect *E. coli* abundance (Blyton et al., 2015). Under high‐intensity conditions, the shift towards anaerobic metabolism may alter the gut environment in a way that could favour not only commensal proliferation but also strains with pathogenic potential (Bielik et al., 2022).

In line with these microbial alterations, we observed upregulated expression of ZO‐1 and IL‐6 in the high‐intensity group. The increase in ZO‐1 expression may reflect a compensatory mechanism in response to epithelial stress or tight junction remodelling. Although ZO‐1 plays a pivotal role in maintaining mucosal barrier integrity, sustained upregulation in this context may signal epithelial vulnerability (Turner, 2009). Increased gut permeability may facilitate the translocation of microbial metabolites such as LPS into systemic circulation, potentially triggering inflammatory cascades (Leppkes & Neurath, 2020).

At the molecular level, we observed upregulated expression of ZO‐1 and IL‐6 in the high‐intensity group. The rise in ZO‐1 expression should not be interpreted as direct evidence of compromised barrier integrity but rather as a compensatory or adaptive remodelling of tight junctions in response to epithelial stress (Shen et al., 2011; Turner, 2009). Although this may help maintain mucosal defence, persistent or dysregulated remodelling could still indicate epithelial vulnerability. The parallel upregulation of IL‐6 supports the notion that high‐intensity exercise elicits systemic inflammatory signalling. IL‐6 is a pleiotropic cytokine: transient elevation can be beneficial for muscle metabolism and repair, but chronic overproduction has been linked to barrier dysfunction, metabolic dysregulation and impaired immune tolerance (Gleeson et al., 2011; Hou et al., 2020; Pedersen & Febbraio, 2008). Mechanistically, intense exercise induces oxidative stress and glycogen depletion, both of which contribute to IL‐6 release and amplify epithelial stress (Fischer et al., 2004; Keller et al., 2005; Xu et al., 2023; Yoon et al., 2024). This interpretation is supported by our regression analysis, which revealed a strong linear association between exercise intensity and both ZO‐1 and IL‐6 expression levels (*R* ^2^ = 0.952 and 0.890, respectively). These findings underscore a dose‐dependent physiological response in which moderate exercise promotes adaptive microbial and immune modulation, whereas excessive training can induce oxidative stress, immune activation and microbial imbalance.

The relationship between exercise intensity and gut health is complex and multifaceted, involving various physiological adaptations and microbial changes. Our findings reveal that moderate‐intensity exercise has a more optimal effect on maintaining gut health. This is supported by various studies that have recognised that moderate‐intensity exercise is beneficial to gut health, especially in maintaining homeostasis, reducing inflammation and increasing the diversity of gut microbiota with better metabolic health (Clauss et al., 2021; Qiu et al., 2023). In contrast, high‐intensity exercise can have both positive and negative effects. For example, high‐intensity exercise is associated with an abundance of certain bacteria, such as *Methanobrevibacter smithii*, which in excess can increase gut permeability and inflammation characterised by gastrointestinal symptoms such as nausea and vomiting (Clauss et al., 2021; Torquati et al., 2023).

It is important to note that this study was conducted in male Wistar rats. Sex hormones play a pivotal role in shaping gut microbiota composition and intestinal barrier integrity through multiple molecular pathways. Oestrogen, for instance, can upregulate the expression of tight junction proteins such as occludin and ZO‐1 via oestrogen receptor‐β signalling, thereby strengthening barrier function (Braniste et al., 2009). In contrast, androgen signalling has been associated with altered bile acid metabolism and immune modulation, which indirectly influence microbial community structure (Ridlon et al., 2013). These hormonal effects intersect with microbial metabolites, such as short‐chain fatty acids and tryptophan‐derived indoles, which further regulate epithelial proliferation, mucosal immunity and oxidative stress responses. Importantly, dysregulation of these hormone–microbiota–barrier interactions may lead to sex‐specific vulnerabilities under physiological stress, such as high‐intensity exercise. Consequently, translating findings from animal models to humans requires caution, as sex hormone levels fluctuate across the lifespan and can markedly alter both microbiota composition and gut barrier responses to exercise‐induced stress (Chella Krishnan et al., 2018; Markle et al., 2013).

Overall, our results align with prior human and animal studies indicating that moderate exercise supports microbial diversity and immune homeostasis, whereas excessive training may contribute to barrier dysfunction and inflammatory stress (Costa et al., 2019; Karl et al., 2017). Maintaining an optimal threshold for exercise intensity appears essential for preserving gut homeostasis and minimising gastrointestinal complications. Beyond exercise, dietary strategies such as prebiotics and probiotics may help mitigate the adverse effects of strenuous training by stabilising microbial communities and strengthening barrier function (Clark & Mach, 2016). Although our findings demonstrate that ZO‐1 expression increased with exercise intensity, the interpretation of this result should be made cautiously. Elevated ZO‐1 mRNA levels may reflect either compensatory upregulation aimed at maintaining tight junction integrity or remodelling processes in response to exercise‐induced stress, rather than a direct indicator of barrier disruption. Since no functional permeability assays (e.g., FITC‐dextran or lactulose/mannitol tests) were performed in this study, we cannot definitively conclude whether the observed molecular changes translated into altered gut barrier function. Future studies combining gene/protein expression analysis with direct assessments of intestinal permeability are warranted to clarify this relationship.

### 4.1 Conclusion

In conclusion, this study provides compelling evidence that exercise intensity plays a pivotal role in shaping gut microbial composition, mucosal barrier integrity and inflammatory responses. Moderate‐intensity exercise promotes a favourable microbiota profile, enhances barrier function and maintains immune balance. In contrast, high‐intensity exercise induces dysbiosis, increases epithelial stress and elevates pro‐inflammatory cytokines such as IL‐6. These findings highlight the importance of calibrating exercise regimens to avoid gut‐related complications whilst maximising systemic health benefits. Future translational studies and integrative omics analyses are warranted to further elucidate the microbiota–host–exercise axis and inform targeted interventions for gut health optimisation.

## AUTHOR CONTRIBUTIONS

Conceptualisation: Nova Sylviana, Nur Faizah Romadona and Imam Megantara. Methodology: Nova Sylviana, Nur Faizah Romadona and Imam Megantara. Software: Putri Karisa and Imam Megantara. Validation: Imam Megantara and Nova Sylviana. Formal analysis: Putri Karisa, Nur Faizah Romadona and Imam Megantara. Investigation: Imam Megantara. Resources: Nova Sylviana and Imam Megantara. Data curation: Putri Karisa, Nova Sylviana and Imam Megantara. Writing—original draft preparation: Nova Sylviana, Nur Faizah Romadona and Imam Megantara. Writing—review and editing: Putri Karisa and Nova Sylviana. Visualisation: Putri Karisa and Nova Sylviana. Supervision: Nova Sylviana and Imam Megantara. All authors have read and approved the final version of this manuscript and agree to be accountable for all aspects of the work in ensuring that questions related to the accuracy or integrity of any part of the work are appropriately investigated and resolved. All persons designated as authors qualify for authorship, and all those who qualify for authorship are listed.

## CONFLICT OF INTEREST

None declared.

## FUNDING INFORMATION

No funding was received for this work.

## Data Availability

The datasets used and analysed for the current study are available from the corresponding author (N.S.) upon reasonable request.
